# Attributes of Perceived Bikeability in a Compact Urban Neighborhood Based on Qualitative Multi-Methods

**DOI:** 10.3390/ijerph16193738

**Published:** 2019-10-04

**Authors:** HaeLi Kang, Dong Ha Kim, Seunghyun Yoo

**Affiliations:** 1 Department of Health Care Policy Research, Korea Institute for Health and Social Affairs, 370 Sicheong-daero, Sejong-si 30147, Korea; hyeri92@kihasa.re.kr; 2 Department of Public Health Sciences, Graduate School of Public Health, Seoul National University, Seoul 08826, Korea; kimdongha@snu.ac.kr; 3 Institute of Health and Environment, Seoul National University, Seoul 08826, Korea

**Keywords:** bikeability, cycling, qualitative research, urban health, active transportation, Korea

## Abstract

Cycling provides opportunities to promote healthy and sustainable cities. However, few studies examine cyclists’ perceived attributes of a bicycle-friendly environment in relation to compact urban contexts. This study explored the attributes of perceived bikeability and urban context related to the cycling experience in Seoul, Korea. Purposive sampling with public recruitment and a snowball technique was used to recruit twenty-two cyclists and three bicycle-related community service providers from a bikeable environment. Qualitative multi-methods, including semi-structured interviews and bicycle tours with a GPS device, were adopted. The main themes of perceived bikeability were derived through thematic analysis. Cyclists perceived the attributes of a bicycle-friendly physical environment as essential components of bikeability. In urban environments where cycling is not yet recognized as the main transportation mode, internal conflict among cyclists and external conflicts between cyclists and other transportation users were evident. A supportive community system included developing an appropriate environment, providing information, and expanding riding opportunities. A bicycle-friendly culture accumulated over a long period influenced the initiation and maintenance of cycling and contributed to a more bikeable community environment. Cyclists’ attitude, behaviors, and perceived environment differed according to purpose. Policy, system, and environmental changes are required to promote cycling in compact urban contexts.

## 1. Introduction

Promoting active transportation is recognized as a strategy to address urban problems caused by car-dependent structures [1]. Cycling, one mode of active transportation, provides environmental benefits by reducing traffic congestion, energy consumption, and carbon emissions, contributing to creating a sustainable city [2]. From a health perspective, cycling promotes an active lifestyle by reducing sedentary behaviors and helps prevent obesity, cardiovascular disease, and diabetes [3]. As urban planners and policymakers agree on the benefits of urban cycling, its promotion has emerged on the policy agenda [4].

To create a bicycle-friendly city, the concept of bikeability is applied to assessing a community’s cycling-related environment. Bikeability is the extent of comfort residents feel when navigating the community via a bicycle [5]. It is determined by the interaction between aspects associated with perceived environment, cycling infrastructure and behavior [6,7]. 

Several studies on bikeability have explored factors influencing cycling [8,9,10] and developed indicators of a bicycle-friendly environment [11,12]. Despite academic discussions on the multidimensional factors of bikeability from a socio-ecological perspective [13,14], existing studies mostly focus on the physical environmental characteristics of bikeability and use objective measurements of the cycling infrastructure [15,16]. This leads to a mismatch between the perceived and objective aspects of bikeability, and there is a risk of missing the community context to which cycling is related [7]. 

Few studies have examined the perceived cycling environment [5,17,18]. These discuss aesthetics, connectivity, convenience, maintenance, and safety as perceived environmental attributes related to cycling, although major attributes differ according to the urban context. Most research on perceived bikeability has been conducted in Western Europe and the US [17,18], and evidence on the cycling experience in the compact urban forms of Asian countries is scant. 

The characteristics of compact cities in Asian countries differ from those of the built environment in Western cities. Seoul, the capital city of South Korea, ranked highest in population density among the Organisation for Economic Co-operation and Development (OECD) member countries, and can be used as a case study to elucidate living in compact urban forms [19]. In the Seoul Metropolitan Area, land uses in the urban core are highly mixed. Furthermore, the building form is high-rise because land-use regulations have been relaxed [20]. Over 90% of the population in Seoul live within a five-minute walk from a bus or metro station, and free transfers between public transportation modes are provided [21]. Since 1998, the Seoul Metropolitan Government has promoted urban policies for active transportation to achieve efficient urban mobility in densely populated and mixed land use areas. Nevertheless, bicycle use as transport in Seoul has only been around 5% since 2012, far lower than European cities [22].

Bikeability is characterized by the lived experience of cyclists and the cycling environment. However, there is limited information on users’ perceived attributes of a bicycle-friendly environment. Furthermore, it is unknown how cyclists behave in compact urban forms and the environmental contexts involved in bicycle use. Therefore, we explored the attributes of perceived bikeability and the cycling experience in the urban context of Seoul.

## 2. Materials and Methods

### 2.1. Setting

This study was conducted in Yangcheon-gu, a southwestern district of Seoul. The overall topography of the district is low-lying land with surrounding rivers. In 2016, the total population was 481,845, with a population density of 26,776 inhabitants/km^2^. Among total households, 72% had one or more children. The central area houses a mixture of cram schools, shopping outlets, restaurants, and large residential high-rise buildings inhabited mostly by middle and upper-income families.

**Figure 1:**
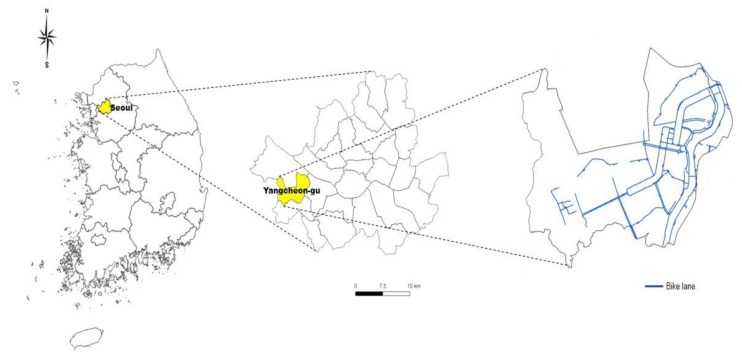
Yangcheon-gu in Seoul.

The city government designated Yangcheon-gu as a bicycle-friendly district in 2004 [23]. The bicycle-friendly environmental features of the district include one-way main streets and riverside bicycle paths. The total length of all-type-bikeways in Yangcheon-gu is 45.15 km, of which 56% are completely separate bicycle paths (Figure 1). Parking facilities for bicycles were constructed at 230 locations to accommodate 12,923 bicycles, including at subway stations, bus stops, and schools. In 2016, bicycle use for transport in Yangcheon-gu was 5.2%, higher than Seoul’s 4.6% [22]. Yangcheon-gu is a representative case of Seoul to explore the context and cycling experience in compact urban forms with a bikeable environment.

### 2.2. Data Collection

We applied qualitative multi-methods, namely in-depth interviews and a bicycle tour to explore the perceived bikeability of the neighborhood. Data were collected from August to October 2016. This study was conducted in accordance with the protocol approved by the Institutional Review Board of Seoul National University (IRB Nos. E1610/003-010 and 1608/001-002).

Purposive sampling of daily cyclists was conducted using a variety of recruitment strategies, including public recruitment at cycling places (e.g., parks, bicycle rental facilities, and bike paths) and a snowball technique with the recruited participants. Interview participants were categorized by the purpose of cycling (transportation or leisure). The interview was saturated [24] with approximately 10 people per category, and an overall number of 22 cyclists (13 transport, nine leisure) participated in the study (Table 1). Semi-structured interviews were conducted to explore the meaning of cycling in urban life, the experience of cycling in a compact urban environment, and the perceived attributes of a bicycle-friendly environment. A brief survey followed to identify participants’ socio-demographic characteristics and cycling frequency.

Each interviewee rode a bicycle with a Global Positioning System (GPS) device to map their usual course of biking in the neighborhood. Photos were taken to specify environmental features and/or situations mentioned in the interview. We met with the participants again to retrieve the GPS and photos and recorded their explanations of the content in the photos. 

Three additional interviews were conducted with public and civic service providers related to promoting cycling in the community. They provided information on the bicycle-related services and policies in the community, which was used to interpret cyclist interviews and describe community context.

### 2.3. Data Analysis

We performed thematic analysis [25] on verbatim transcripts of audio-recorded interview data. After reading the transcripts repeatedly and becoming familiarized with the data, we derived 108 codes for the concepts and characteristics related to bikeability. Themes were derived through comparing, matching, and categorizing the codes.

Spatial data were used to analyze cycling behavior, and “where” and “how” cycling-related factors were distributed. The GPS records and photos collected through the bicycle tour were synthesized to Google Maps [26]. Participants’ paths were color-coded to identify the bikeway used. The bike icon and a brief description were attached to the places the pictures were taken. This mapping enabled the identification of patterns and characteristics of cycling in daily life and perceived bikeability. Specifically, visual and spatial data provided numerical information on participants’ behavior and identified their perceived bikeability in a real environment. The comparison and analysis of information collected through qualitative multi-methods reinforced the trustworthiness of the study through triangulation [27].

## 3. Results

The attributes of perceived bikeability were derived from the following four themes: (1) a bicycle-friendly physical environment, (2) supportive community system, (3) cultural influence, and (4) conflicts over cycling. The purpose of cycling induced differences in perceived bikeability and cycling behavior.

### 3.1. “Bicycle-Friendly” Physical Environment

Cyclists in this study reported cycling safety by avoiding congestion, convenient usability of cycling infrastructure, and pleasantness of the neighborhood environment as attributes characterizing a bicycle-friendly physical environment. All participants felt “safe” when bikeways were separate from motor or pedestrian traffic. In the community, most destinations for transportation cycling were concentrated in the central area, which comprises mixed traffic streets. In particular, transportation cyclists felt anxiety and fatigue when cycling in the central area and a designated cycling space was needed to improve their safety. However, public service providers responded that the street design for mixed land use was inevitable in the central area and there were institutional and spatial limitations on expanding bicycle-only spaces.

**Figure 2:**
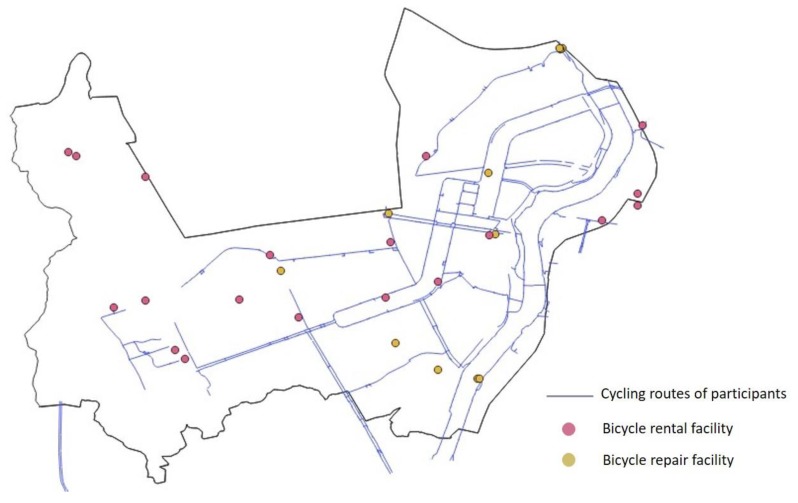
Spatial pattern of cycling behaviors and infrastructure.

**Figure 3:**
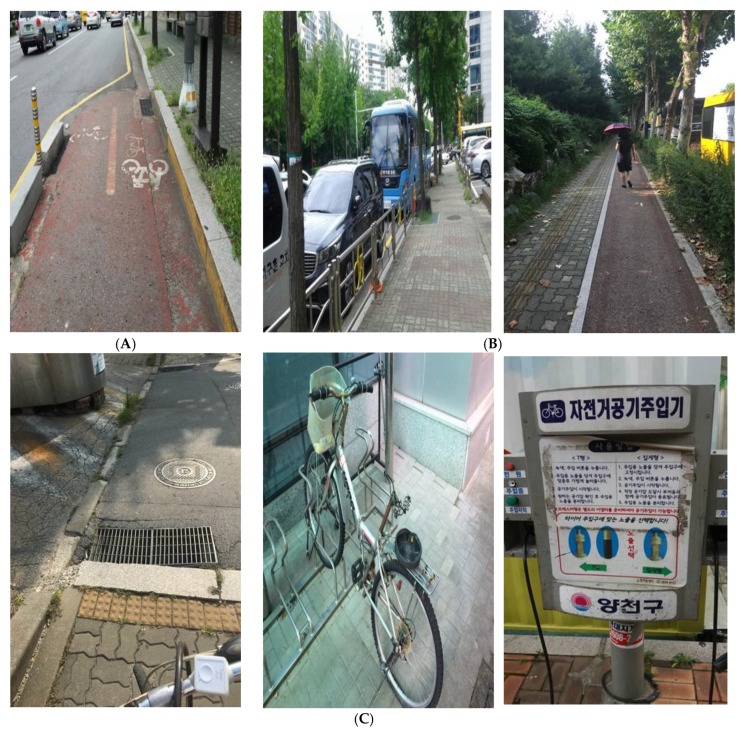
Factors that hinder bikeability. (**A**) “Bicycle-friendly” physical environment—Disconnection of cycle tracks. (**B**) Conflicts over bicycle use: (left)—Illegal parking; (right)—Pedestrian walking on bikeway. (**C**) Supportive community system: (left)—Poor management of road; (center)—A bicycle with a saddle stolen; (right)—Bicycle management equipment with insufficient instruction.

“Convenience” of the cycling infrastructure was an attribute which influenced cycling behavior and experience. Most participants defined convenience as useful and usable infrastructure related to overall cycling behavior such as bicycle riding, parking, maintenance, and taking a break while cycling. Most photo data by the participants (35 of 48 photos, 72.9%) featured the connectivity and accessibility of bikeways as examples of a convenient cycling environment (Figure 3A). Furthermore, highly accessible and well-maintained bicycle repair facilities, bicycle parking facilities, and rest benches were highlighted as assets to facilitate cycling. However, the spatial behavior maps from the bicycle tours showed areas where cycling behavior patterns and cycling infrastructure were mismatched (Figure 2).

“Pleasantness” was an attribute related to the emotional benefits of cycling. Motivations for leisure purposes included emotional emancipation and stress reduction. Most leisure cyclists preferred cycling in spacious parks or green spaces. Condensed community design, including the distribution of amenities close to residential and mixed land use areas, was also identified as a physical environmental factor driving cycling for fun. However, interesting places to cycle such as the main street area posed safety issues because their aesthetic appeal caused high congestion. Furthermore, natural environmental aspects such as the weather, landform, and air quality affected the perceived pleasantness of the neighborhood environment. Specifically, fine dust hindered participants’ enjoyment of outdoor activities and use of bicycles.

All participants identified a bicycle-friendly physical environment as a multi-attribute concept related to various environmental factors such as land use, transportation, the street environment, green spaces, and natural environment. On the other hand, all service providers focused only on attributes related to cycling infrastructure, showing a narrower scope. 

### 3.2. Conflicts over Cycling

All transportation cyclists experienced conflicts with pedestrians and motorists in the community, where biking is not yet considered a major mode of urban mobility (Figure 3B). Some pedestrians, ignorant or unaware of bicycle lanes and roads, were often in cyclists’ way. Jaywalking and pedestrians using smart phones while walking increased the risk of accidents, increasing cyclists’ anxiety. This involved the attitude of pedestrians familiar with the mixed traffic streets and high street density. The greatest conflict was with motorists. Drivers unhappy about sharing roads with cyclists reacted aggressively by honking or pushing cyclists to the shoulder of roads. Motorists’ lack of awareness of “cycling as part of the community traffic system” resulted in illegally parking vehicles in bikeways and driving in a way inconsiderate of cyclists. This increased cyclists’ level of discomfort and intimidation and discouraged participants from riding bicycles.

Most participants also experienced internal conflicts. They wanted to be considerate of cyclists; however, they realized that they become impatient and agitated when they felt that cyclists hindered their walking or driving. Internal conflict also occurred regarding protective gear. While safety was a priority among cyclists, they did not wear a helmet or other protective gear because wearing such equipment is neither fashionable nor comfortable. The perception that cycling in the neighborhood in everyday life is not as serious and dangerous as cycling as a sport also contributed to not using personal protective equipment.

### 3.3. Supportive Community System

The limitations of individual efforts to increase bicycle use and need for a community system were discussed. The supportive community system defined by participants means creating and managing policies that support cycling at the organizational and societal level and expanding cycling opportunities in the community. Here, cyclists and service providers agreed that a priority policy area was to create and manage the physical environment for cycling (Figure 3C), as described in the theme of a “bicycle-friendly” physical environment. 

Building an anti-theft system was also emphasized. Bicycle theft was a major concern, greatly influencing the usage and maintenance of bicycles in the community (Figure 3C). This concern discouraged cycling, as transportation cyclists were reluctant to park their bicycles in public places. Parking bicycles inside residential buildings caused inconvenience and aesthetic disturbance and formed a negative perception of cycling among residents. This resulted in a high demand among cyclists for institutional measures to prevent bicycle theft and penalize perpetrators. Public service providers responded that the indifference to neighbors and weakening of social trust increased bicycle theft, even though a bicycle registration system was operating in the community. 

Information provision related to proper and safe cycling was highlighted. Participants with children contended that education was needed on how to ride a bicycle safely in accordance with related rules and regulations. All participants, except those who have learned to cycle through public services, learned how to ride in childhood from family members, and often lacked information on traffic rules and related safety regulations. Cyclists had a high demand for information on bicycle maintenance (Figure 3C), cycling-related traffic regulations, and available bicycle-related services. Cyclists mentioned the low utilization of public services related to cycling, because of a lack of public relations. However, service providers thought there was sufficient publicity regarding cycling-related services.

The public bicycle rental system and incentive policies were discussed regarding expanding opportunities to cycle. All participants thought a public bicycle rental system would improve access to bicycles. Some older participants, however, were concerned that using the bicycle rental system would be complicated and difficult for the elderly and that introducing it without institutional and spatial improvements would increase traffic congestion and accidents. On the other hand, some stores in the community were using a strategy of providing eco-friendly points that consumers could use in lieu of money when they cycled to the store. Housewives were aware of this strategy and expected it would spread throughout the community. A civic service provider indicated the need for partnerships between public, private, and civic groups to spread economic incentive strategies.

### 3.4. Cultural Influence

A bicycle-friendly culture was recognized as a community strength to promote and maintain bicycle use. Cycling culture among family, peers, and the community played a key role in starting and sustaining cycling. Bicycle-friendly family culture was a driving force to start and continue cycling. Fathers played a vital role in instilling the culture as the main cyclist and cycling teacher in the family. Children raised in a bicycle-friendly family had fewer challenges with cycling again after not doing so for a while. Conversely, those without family support encountered later opposition from family and friends to ride bicycles. To strengthen family support for bicycle use, public service providers were planning to build a bicycle safety education center for children and run bicycle classroom programs for fathers and their children.

The influence of peer culture was most noticeable among adolescents. Teenagers considered bicycles a means of school transportation and of socializing with friends. They used bicycles to go to school with their classmates, or to cruise along the river afterward. As cycling becomes part of the youth culture, community efforts are being organized to support adolescent cycling. Recently, private bicycle repair shops contracted with a public organization and began providing students with services. They regularly visit schools to repair bicycles, and some schools increased the number of bike shelters based on students’ demands.

A community culture wherein people of all ages use bicycles influenced residents’ perception of cycling as easy and feasible for everyone. A social environment that positively evaluates cycling gave community members a positive image of cyclists being “lively, healthy, and diligent.” Reciprocally, the wide use of bicycles and positive attitude toward cycling reinforced the community culture and urban policies that encourage it. Table 2 shows perceived bikeability according to the themes and categories. Table 3 provides participants’ quotations according to themes.

### 3.5. Differences in Perceived Bikeability According to Purpose of Cycling

Perceived bikeability and its attributes differed according to the purpose of cycling. Safety was an important environmental attribute regardless of the purpose of cycling. Convenience and accessible cycling environment were salient for transportation cyclists. Interesting sights, green spaces, and places to rest were important factors for leisure cyclists. The location and type of bicycle parking facility near the destination were more significant to transportation cyclists than to leisure cyclists, who seldom considered parking.

“Now I commute by bicycle. Because the transportation at Shinjung intersection… I have to go to Shindorim and from there transfer once again. So surprisingly, that way it took more time to go to work. It took me 15 minutes longer (than by bicycle). If the environment hadn’t improved conveniently for cycling, I still (sic) wouldn’t have used a bike to commute.”

(Transport, 50s, woman)

“I ride around [the] neighborhood. A turn around as following this bike road by riverside, when [I have] the day off, I ride (a bike) just like that pretty easily. (…) The riverside is a place for mental relaxation and my favorite place to ride to. ((laugh))”

(Leisure, 60s, woman)

Regarding the social factors of bikeability, transportation cyclists highlighted conflicts with motorists and pedestrians. The influence of peers and family culture was not as great in this group, since they tended to ride bicycles alone.

“There is a clear distinction between bike path and sidewalks, but it’s annoying for some pedestrians to ignore the sign and walk on the bike path.”

(Transport, 20s, woman)

“I only use bicycles for transportation. [the] Cycling commute save[s] me from the fatigue of public transport. I worry that if people ride too many bicycles, the cycling environment will be crowded.”

(Transport, 30s, man)

**Figure 4:**
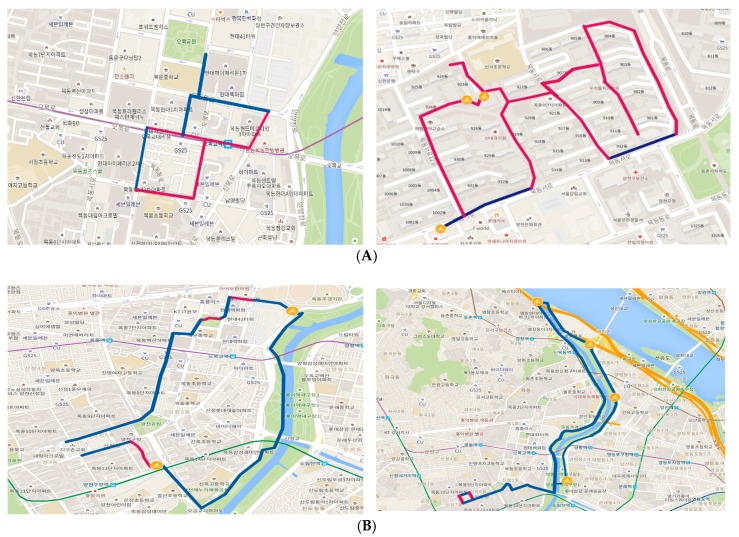
Examples of cycling behaviors by purpose of cycling. (**A**) Cycling for transport: (Female, 20s), Travel distance: 2.5 km, Average speed: 8.0 km/h, Cycling for commuting (working)—(left); (Male, 60s), Travel distance: 3.3 km, Average speed: 7.0 km/h, Cycling for running errands—(right). (**B**) Cycling for leisure: (Female, 20s), Travel distance: 6.7 km, Average speed: 9.7 km/h, Cycling for leisure around the park and neighborhood—(left); (Male, 40s), Travel distance: 14.7 km, Average speed: 12.7 km/h, Cycling for leisure to follow cycle tracks along the river—(right). Blue line: Bikeway, Red line: Not bikeway, Bicycle icon: Photo spot. The information in parentheses shows sex and age of participant.

Cycling behaviors also varied according to the purpose of cycling. Transportation cyclists aim to reach their destinations, and thus use familiar roads (Figure 4A). Leisure cyclists try new routes to visit new places (Figure 4B). They also want to ride with family or other people, unlike transportation cyclists, who ride alone. In the bicycle tour of this study, leisure cyclists rode longer distances at higher average speeds (5.5 km, 9.4 km/h) than transportation cyclists (3.5 km, 7.7 km/h). Transportation cyclists rode equally on bikeways and vehicle roads and used small streets and alleys to get to their destinations. Many leisure cyclists used bikeways. Transportation cyclists had a relatively lower average speed than leisure cyclists, because they had to stop or slow down for pedestrians or cars. Transportation cyclists were mostly commuters and shoppers with fixed destinations that they reached by riding on various types of roads. Leisure cyclists chose destinations on or near bikeways that were easy and pleasant to reach by bicycle.

## 4. Discussion 

We explored the perceived environment and urban neighborhood context for cycling in Seoul. Using a multi-method qualitative approach, descriptive and spatial information about cyclists’ perceptions, behavior patterns, and spatial contexts was collected. Spatial behavior data from the bicycle tours enhanced the qualitative description.

Safety was a common environmental attribute in studies on bikeability [28,29,30,31,32] and was identified as the most prominent attribute in this study. All study participants reported experiencing one or more safety threats and high fatigue on mixed traffic roads. A comparative European study [33] showed that traffic congestion in mixed traffic infrastructure increases cyclists’ fear of traffic and results in cycling avoidance. These suggest that threats to cycling safety caused by urban congestion may be a critical deterrent to bicycle use in compact urban forms. In compact and dense neighborhoods like that in our study, urban planning focused on separating the bike paths without increasing traffic congestion as an effective means to reduce the risk of cycling accidents. According to the concept of Woonerf [34], a home zone [35] and car-free zone can be useful for ensuring a bicycle-friendly space in the context of compact urban forms. Advocacy to ensure a bicycle-only space is also an important consideration in the introduction of this concept.

Convenience was also identified as a key attribute of perceived bikeability. Previous studies on bikeability focused on the accessibility and connectivity of bikeways in measuring the convenience of the cycling environment [36,37]. Similarly, cyclists in this study noted how the good connectivity and accessibility of bikeways influenced their intention to cycle. Furthermore, the ease of bicycle storage and repair and accessibility of rest facilities were important factors perceived as part of a convenient cycling environment. However, the results of the spatial behavior maps showed an imbalance between cyclists’ demand and the supply of cycling infrastructure. This may affect the gap between the convenience of the perceived and objective environment. It is hoped that these findings will advise the assessment of cycling behavior and the spatial pattern of the cycling environment in the development of a cycling promotion master plan.

Pleasantness was related to psychological aspects of the cycling experience, which was affected by the built environmental and natural environmental factors. Urban green space was a stress-relieving place for cyclists and was acknowledged as a key element of a pleasant cycling environment. Barton and Pretty [38] reported that green exercise such as cycling in green spaces enhances positive feelings and provides psychological stability. Interesting places with many attractions were also identified as a pleasant cycling environment. However, our finding suggests urban disorder caused by attractive public spaces can adversely endanger the safety of cyclists. The previous systematic review about the double-edged impacts of the built environment on health supports our results [39]. Our participants mentioned attitudes to cycling were affected by natural environmental factors such as hilliness, air quality, and weather. Specifically, fine dust in the air was identified by participants of all ages as a critical factor hindering the use of bicycles and has emerged as a major health risk factor in recent urban health studies [40,41]. In compact neighborhoods where cycling environments interact with other urban environments, intersectoral and interdisciplinary collaboration is essential in order for pleasant cycling to address issues beyond the cycling infrastructure.

Previous studies noted active transportation as an alternative for automobile use for urban mobility [42,43]. However, there is little mention of problems arising from introducing active transportation. Our findings regarding the conflict over cycling indicated multiple types of conflicts in the process of active transportation in compact urban forms. Speeding by automobiles and bicycles, parking violations, not yielding to bicycles or pedestrians, walking on bike paths, and jaywalking are examples of external conflicts. Cyclists also had mixed feelings about the attitude toward cycling in compact urban forms. These conflicts are byproducts of the compact urban structure for mixed land use and lack of community awareness of cycling as a main mode of transportation. To instill cycling as the main mobility mode in compact urban forms, environmental and communication interventions may be required to address these conflicts. Accordingly, legal guidelines and cycling insurance should be established.

A supportive community system was considered essential for shifting from driving to cycling. Such a system includes anti-theft measures, bicycle facilities management, information sharing, bicycle sharing programs, and incentive programs to encourage cycling. Our findings support the need to build a bicycle-friendly system beyond individual efforts [44,45,46,47,48]. Bicycle theft is a main challenge deterring people from owning and using a bicycle for transportation [47,48]. Bicycle theft in the study area was the combined result of physical environmental limitations and lack of social capital. This suggests community intervention strategies to improve the physical and social environment may prevent and control bicycle theft. Scheepers et al. [49] reported that establishing a bicycle sharing program was an effective intervention for shifting from car to active transport. The Seoul citywide bicycle rental system was launched during the study period, increasing access to public bikes in the community. However, unequal access to bicycle infrastructure and information could occur when promoting active transportation. Particularly, online-based cycling information sharing and unmanned bicycle rental service were a big obstacle for the elderly, who often cannot freely use smart phones. Lee, Sener, and Jones [50] contended that overlooking the impacts of cycling equity resulted in the unequal distribution of the benefits of active transportation. Therefore, a health equity assessment is needed for equitable active transportation planning. 

Previous studies on bikeability focused less on social environmental factors [18]. Ducheyne et al. [51] and Emond and Handy [52] reported the role of family members and friends in getting children and adolescents to cycle as a social environmental factor. Our findings revealed cultural influence also affected people in other age groups. This influence was cumulative and spread within the community. Furthermore, a bicycle-friendly culture was a prominent factor lowering barriers to start riding bicycles. This is aligned with the results of health promotion studies confirming that a health-oriented culture contributes to changing the environment and policy for health promotion in the community [53,54]. These findings highlight a bicycle-friendly culture based on community involvement as a sustainable driving force for environment, policy, and system changes to promote cycling for urban health.

The cyclists in this study emphasized distinct aspects of bikeability according to the purpose of cycling. Similarly, Ma and Dill [7] reported that the subjective and objective environment varied depending on the purpose of cycling. The results of bicycle tours in our study illustrated that, depending on the purpose of cycling, perceived bikeability influenced cycling behaviors and attitudes. In communities with infrastructures that accommodate different types of cycling, the strategies to promote it may be tailored to cycling purposes. 

Another contribution of this study is that it identified multiple aspects of bikeability. In line with a socio-ecological framework, bikeability is more than a one-dimensional characteristic of the physical environment. It encompasses the perceived attributes of the natural and built environment conducive to cycling, attitude, and behavior of people in the community regarding cycling and a community system and culture. Bikeability is a comprehensive concept with multiple interrelated domains associated with their likelihood and cycling skill level. Few other studies have addressed the notion of a community system and culture that supports cycling. This is because most previous research has focused on measurable factors to develop and evaluate indicators for the physical environment.

Although combining spatial and descriptive information in a qualitative study is a novel approach [55,56], methodological discussion on analytic approaches to synthesize such information is limited. Additionally, season and weather at the time of data collection might have influenced the bicycle tour. Future research should employ repeated measurements, participant observation, guided tours, and/or advanced mapping techniques. Despite these limitations, our efforts to apply multiple data collection methods and include various stakeholders in a qualitative study on bikeability have elucidated the attributes of perceived bikeability in the context of compact urban forms.

## 5. Conclusions

This study contributes to active living research by suggesting the attributes of perceived bikeability in a compact urban neighborhood. The attributes of perceived bikeability are multifaceted, and influenced by the purpose of cycling and the context of compact urban forms. Strategies to promote urban cycling need to consider the lived experience of cyclists and urban contexts of their lives. Interdisciplinary collaboration is required to build a bicycle-friendly city that addresses the multiple aspects of bikeability.

Qualitative multi-methods are useful for understanding how behavior and the environment interplay. They also enhance the trustworthiness of the findings, which compared and synthesized several types of information to provide a rich and in-depth understanding of the topics and communities of interest. From a pragmatic perspective, application of qualitative multi-methods can provide substantial new leverage for addressing urban health topics.

## Figures and Tables

**Table 1:** Participants’ socio-demographic characteristics.

Cyclists	N = 22	Service Providers	N = 3
Purpose of cycling		Sex	
Transport	13	Female	1
Leisure	9	Male	2
Sex		Type of service sector	
Female	11	Public	2
Male	11	Community organization	1
Age in years		Type of service	
16–19	6	Policy	2
20–39	7	Advocacy	1
40–59	5		
60+	4		
Years of Residence			
1–9	6		
10–19	9		
20–29	4		
30+	3		
Type of Residence			
Apartment complex	16		
Single-family house	3		
Multiplex	3		

**Table 2:** Themes and categories of perceived bikeability.

Theme	Category	
“Bicycle-friendly” physical environment	Cycling safety by avoiding confusion	Degree of traffic congestion, traffic volume and speed of car, mixed land use, bikeway width, paving, and marking, and lighting
	Convenient usability of cycling infrastructure	Bikeway connectivity and accessibility, bicycle parking facility, bicycle repair facilities, and rest facilities
	Pleasantness of the neighborhood environment	Air quality, topography (slope), weather, community amenities, greenery, interesting sight, and open space
Conflicts over cycling	Conflicts with pedestrians and motorists	Speeding of automobiles and bicycles, parking violations, not yielding to bicycles or pedestrians, walking on bike paths, and jaywalking
	Internal conflicts among cyclists	Double standard for cycling, and non-preference for protective equipment
Supportive community system	Building and management	Building and managing the physical environment and a system for bicycle theft and management
	Providing information	Systematic safety education, provide information related to cycling, publicity, and communication
	Expanding opportunities	Public bicycle rental system and incentive policies for cycling
Cultural influence	Family culture	Formation of intimacy for bicycles, and Learning to ride a bicycle by father
	Peer culture	Popularity of bicycle commuting to school, and socializing with friends through bicycle
	Community culture,	Positive community awareness and attitudes of cycling

**Table 3:** Quotations by themes.

Theme	Category	Quote
“Bicycle-friendly” physical environment	Cycling safety by avoiding confusion	“I’ve tried to ride a bicycle in the neighborhood, but it was too hard because of the road (condition). Bikeway and sidewalk were combined together, and I shouldn’t use the road either; besides, the bikeway is disconnected in the middle of the way like that.” (Transport, 50s, woman)
	Convenient usability of the cycling infrastructure	"There are also some inconveniences in (the) traffic situation, and I think it can be a bit risky for biking due to such heavy traffic. (∙∙∙) It is really dark when I come into the apartment complex—not a few lights, but a lot of trees. So, when I ride (a bike) at night, I use the path with many streetlights, even if there are many cars. It’s because If I go through the apartment complex, I cannot really see the areas being dug.” (Leisure, 30s, man)
	Pleasantness of the neighborhood environment	“When I go by Yangcheon Park, I see many things going on—for example, some mid-aged women doing exercises inside the park. It’s fun to watch such thing while riding a bike. A market is also opened in the area and I watch that as well.” (Transport, 10s, man)
Conflicts over cycling	Conflicts with pedestrians and motorists	“(Conflicts with pedestrians) I mean, there’s a bicycle road right in front of my house but with sidewalk. Then, there are so many people who do not recognize the bicycle road and walk on.” (Leisure, 20s, man) “(Conflicts with car drivers) Cars just push the bicycles ahead. Yeah, it’s just so dangerous for the bike users. Originally, bike riders use the side road, but sometimes there are obstacles in the middle of the way then bikers should break into the car in order to keep going. However, the cars hate that and so just push them away.” (Transport, 60s, man)
	Internal conflicts among cyclists	“[From a car driver’s point of view] Annoying, to be honest. Yeah, but I cannot say anything about it. I just circumvent (the bikes). Each time, all points of views are different: as a pedestrian when I walk, as a biker, and as a driver.” (Transport, 60s, man)
Supportive community system	Building and management	“It’s about the facilities, bike infrastructures. Above all, bicycle roads, installed air injectors, and bike racks, these kinds of things. These parts, it is important that we maintain the facilities better in use.” (Service provider A) “(transfer with a bicycle) I almost did not (ride a bike). Because I am afraid my bike being robbed ever since I bought a new bicycle. Anywhere you tie it up, soon… (it’s disappeared)” (Leisure, 20s, man)
	Providing information	“(About public bicycles) Its biggest problem is lack of promotion; so, I wonder anyone’s using it.” (Leisure, 30s, man)
	Expanding opportunities	“(About public bicycles) That bike at first, I thought it just takes up a lot of space and results the tax lost, but no. I see many people using it. So, I wondered where the idea came from which is great. Empty means people use them that much.” (Transport, 50s, women)
Cultural influence	Family culture	“I got to ride bicycle(s) often because of my dad who has liked bicycling. So, I’ve ridden many times to Han River, and most time I ride with my dad during vacation.” (Transport, 10s, woman)
	Peer culture	“Yes, there are some friends who have bought new bikes these days: a friend who did not ride (a bike) and almost did not exercise, and friends hanging out here that used to not ride at all, but instead playing (computer) games or billiards.” (Transport, 10s, man)
	Community culture	“(Riding a bicycle in my neighborhood) is not awkward at all. I don’t know it’s because I’m used to this atmosphere, but I just think, ‘yeah riding bicycles,’ when I see the people in 30s or 40s riding. When I go, however, another neighborhood, it seems a bit awkward even though riding is the same. Being awkward there and being accustomed here…” (Transport, 10s, man)
